# Giant thoracic schwannoma presenting with abrupt onset of abdominal pain: a case report

**DOI:** 10.1186/1752-1947-3-88

**Published:** 2009-10-30

**Authors:** Isaac Yang, Elena Paik, Nancy G Huh, Andrew T Parsa, Christopher P Ames

**Affiliations:** 1 Department of Neurological Surgery, University of California San Francisco, San Francisco, CA 94143, USA

## Abstract

**Introduction:**

Giant intradural extramedullary schwannomas of the thoracic spine are not common. Schwannomas, that is, tumors derived from neoplastic Schwann cells, and neurofibromas represent the most common intradural extramedullary spinal lesions. We report the case of a patient with a giant thoracic schwannoma presenting unusually with acute abdominal pain and with delayed neurological impairment.

**Case presentation:**

A 26-year-old Hispanic man with no previous medical problems presented with acute periumbilical pain. After extensive work-up including an exploratory laparotomy for appendectomy, magnetic resonance imaging scans of the lumbar and thoracic spine revealed a giant intradural extramedullary thoracic schwannoma within the spinal canal posterior to the T9, T10, and T11 vertebral bodies. Magnetic resonance imaging signal prolongation was noted in the spinal cord both rostral and caudal to the schwannoma. The patient underwent an urgent laminectomy from T8 to L1. After sacrificing the T10 root, the tumor was removed en bloc. Postoperatively, the patient improved significantly gaining antigravity strength in both lower extremities.

**Conclusion:**

The T10 dermatome is represented by the umbilical region. This referred pain may represent a mechanism by which a giant thoracic schwannoma may present as acute abdominal pain. Acute, intense abdominal pain with delayed neurologic deficit is a rare presentation of a thoracic schwannoma but should be considered as a possible cause of abdominal pain presenting without clear etiology. Although these lesions may be delayed in their diagnosis, early diagnosis and treatment may lead to an improved clinical outcome.

## Introduction

Schwannomas, that is, tumors derived from neoplastic Schwann cells, and neurofibromas represent the most common intradural extramedullary lesions, accounting for approximately 30% of all nerve sheath tumors in adults [1,2]. These soft-tissue tumors generally do not grow greater than 8 cm in diameter [3]. Giant schwannomas, as reported in this case, differ from normal schwannomas because of their substantial size [4]. Rare giant schwannomas in the spine are most often discovered in the cauda equina because of the ability of the tumor to grow unnoticed to large proportions without causing any irritation of the nerve roots [5], but have also been found in various other areas on the extremities of the body [3,6].

Schwannomas commonly appear radiographically as 'dumbbells' that traverse across the dural lining, and comprise about 25% of all spinal tumors [7-10]. These tumors are usually lobulated, encapsulated, and well demarcated [11] and can occur in diverse locations including the thoracic region. Thoracic schwannomas typically manifest initially in the fourth or fifth decade of life without a higher incidence in either gender [11,12]. We report the case of a patient with a giant thoracic schwannoma presenting with intense periumbilical pain and with delayed neurological impairment.

## Case presentation

A 26-year-old Hispanic man with no previous medical problems, presented with chronic abdominal pain that he had been experiencing for 2 years. Approximately 1 year before presentation at our facility, he underwent a peritoneal biopsy which did not reveal anything abnormal. Over several days, the patient developed acute periumbilical pain and was admitted for work-up of this acute presentation of abdomen pain. The patient had not had this acute periumbilical pain before but had experienced chronic abdominal shooting pain for up to 2 years previously. He reported no cramps, no nausea, no vomiting, and no migration of the pain. The periumbilical pain was a new onset, abrupt continuous sharp feeling of pain that was different from his chronic abdominal discomfort. On physical examination, he was afebrile and had a soft and minimally tender abdomen. Although he had periumbilical pain, it was not exacerbated by palpation, and there was no rebound and no guarding on examination. His bowel sounds were normal. His laboratory tests revealed a normal white cell count, liver function, and chemistry panels.

The work-up for this abrupt onset of acute abdominal pain was evaluated by upper gastro-intestinal (GI) endoscopy, radiographic upper GI series, and finally a surgical exploratory laparotomy which revealed no apparent bowel pathology and during which an appendectomy of a grossly normal appearing appendix was performed. All of these procedures revealed normal anatomy of the peritoneum and abdominal organs. During the week of admission for work-up of this abrupt onset of acute abdominal pain, the patient developed sudden, severe lower extremity weakness and an abrupt lower extremity decrease in sensation.

Within 48 hours of onset, the patient's physical examination demonstrated a flaccid paraplegia of the lower extremities. There was decreased sensation to light touch and joint position sense below approximately T10. Pinprick and temperature sensation were diminished below the T9 dermatome. The patient did have some minimal distal lower extremity muscle function intact without antigravity. His rectal tone was mildly decreased and his lower extremity reflexes were hyper-reflexive with an up-going Babinski reflex.

**Figure 1:**
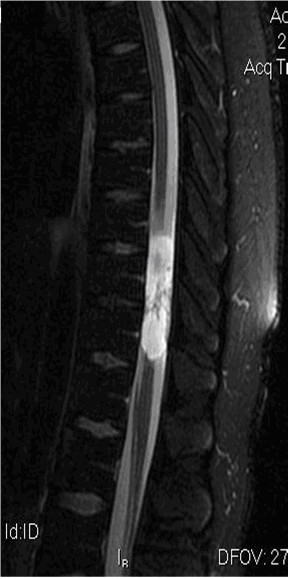
**Sagittal T2-weighted thoracic magnetic resonance image**. Note intradural extramedullary lesion at T9-T11 (arrow).

**Figure 2:**
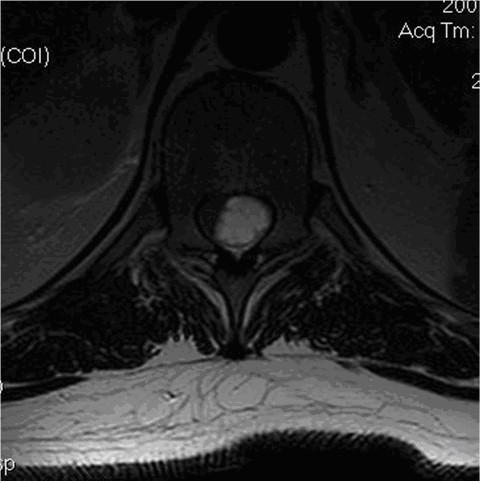
**Axial T2-weighted thoracic magnetic resonance image**. Note intradural lesion at T10 (arrow).

Emergency magnetic resonance imaging (MRI) scans of the lumbar and thoracic spine revealed an intradural extramedullary mass within the spinal canal posterior to the T9, T10, and T11 vertebral bodies. The mass was multicystic and well circumscribed with contrast enhancement of the periphery (Figure 1 and Figure 2). The MRI scan also showed that the spinal cord was compressed by the mass and demonstrated T2 prolongation in the spinal cord portions that were immediately superior and inferior to the mass.

The patient underwent an urgent laminectomy from T8 to L1. Intra-operatively, it was noted that the spinal cord was severely compressed most markedly at T10. After sacrificing the T10 root, the tumor was removed en bloc. With the gross appearance of a schwannoma intra-operatively, the histologic tissue analysis confirmed this diagnosis with observation of spindle-shaped Schwann cells and elongated nuclei consistent with a schwannoma tumor. There were no malignant or atypical features on the tissue histologically on pathologic analysis. Postoperatively, after resection of this giant thoracic schwannoma, the patient improved significantly gaining antigravity strength in his lower extremities.

## Discussion

Patients with nerve sheath tumors generally follow an indolent clinical course related to slow tumor growth; this gradual displacement of the spinal cord causes symptoms proportionally less severe than expected from the tumor size. Back pain without other neurological signs is the most common complaint from these patients [7]. The delayed and rapid onset of abrupt lower extremity weakness experienced by our patient is rare in the reported literature for patients with thoracic schwannoma [11].

The presentation of symptoms can be extremely diverse depending on the areas of the spine affected by nerve cord tumors [13]; tumors can be located in the cervical region, thoracic region, or lumbar region with numerous studies showing preferences for all three areas [7,11]. However, nerve sheath tumors are most often reported in the low thoracic and thoracolumbar regions with 50% of the cases reported below T8 [7,11]. Typically, the growing tumor in any of these areas creates a sharp, stabbing pain due to the compression and resultant irritation of the nerve root in the foramen [13].

In spinal lesions between T8 and T12, the patient is likely to feel pain on the surface of the abdomen or within the abdominal cavity [14]. The corresponding dermatome to T10-T12 encompasses the umbilical region and lower abdominal region as well as both right and left flanks. In addition, the stomach, small intestine and large intestine, including the appendix, are innervated by these same levels. It has been well documented that disorders of the spine or stimulation of spinal structures can cause referred pain to other areas innervated by the same spinal segments. Jorgensen and Fossgreen [15] suggest somatovisceral reflexes, which may be activated by nerve root irritation at the intervertebral foramina, as a connection between abdominal pain and abnormalities in the lower thoracic spine.

In a report of two cases of patients with spinal disease, Jooma and Torrens [14] presented a 73-year-old woman with an 8-year history of lower back pain that radiated to the flank and right lower quadrant of the abdomen. After undergoing an appendectomy to remove a normal appendix and a negative intravenous pyelogram, subsequent anal sphincter failure necessitated a neurological assessment which allowed the correct diagnosis of an intradural extramedullary tumor at T10 to be made.

Neinstein reported a similar case in an 18-year-old man who presented with a 1.5-year history of left lower quadrant abdominal pain, left-sided flank pain and left lumbar back pain radiating to the buttocks. The correct diagnosis of a T11 schwannoma was reached after almost 1 year, upon which he underwent a T10-T11 laminectomy and postoperatively, recovered sufficiently to return to normal activities [16].

Cox and Alter report a challenging diagnosis in a 30-year-old man who presented with unilateral abdominal and back pain. Despite multiple evaluations, the cause of the symptoms was not identified until 11 months after the beginning of the symptoms, when an MRI scan showed an intradural extramedullary schwannoma at the T10 level [13].

This case report presents a giant thoracic schwannoma as a difficult and challenging diagnosis for acute abdominal pain with delayed neurologic findings. Giant thoracic schwannomas rarely present in this manner because most schwannomas become significantly symptomatic once the spinal cord is compromised. This usually occurs before the schwannoma can grow to a giant size. Hence most neoplasms are detected before they grow to such large sizes in the thoracic spinal canal. Furthermore, the evidence for referred pain between the abdominal viscera and the innervations of specific spinal cord levels indicates a possible mechanism by which a slow-growing giant neoplasm could present with the abrupt presentation of an acute abdomen.

Patients with low thoracic or high lumbar lesions with unusual presentations and late neurologic symptoms may experience a significant delay in diagnosis and treatment of their tumor. In a retrospective study of 42 patients with spinal schwannomas, Asahara and Kawai [7] report that most delays in diagnosis occur for lumbar schwannomas but early diagnosis and intervention improved the clinical outcome for all schwannomas.

Reaching the decision to test for schwannomas when faced with unusual symptoms is problematic, but once a peripheral nerve sheath tumor is suspected, the testing is relatively straightforward. The advent of computed tomography (CT) and MRI has decreased mortality and morbidity rates and these technologies are still the main methods for diagnosis and detection of the lesions. MRI is the method of choice over CT since it has been proved to be equal to or better than CT in analyzing this type of tumor [2,7,11,13,14].

Although MRI has improved the diagnosis of spinal cord tumors, the precise histologic diagnosis cannot be made with radiographic studies alone. Tissue diagnosis is required to establish diagnosis in most spinal cord tumor cases.

Finally, because of the propensity for neurofibromatosis type 2 patients to develop schwannomas, genetic testing for the neurofibromatosis mutation as well as merlin, a tumor suppressor gene localized on chromosome 22q12, should be performed in patients diagnosed with spinal cord schwannomas. Malignant lesions are more commonly associated with neurofibromatosis type 1 and should be considered in solitary tumor cases if other cutaneous manifestations are present. Neurofibromatosis type 2 is more commonly associated with multiple benign tumors.

## Conclusion

Thoracic schwannomas can present with acute onset of abdominal pain without clear etiology and without neurologic deficit. We report an unusual case of a patient with a giant thoracic schwannoma, who presented with abrupt and acute onset of abdominal pain over several days and underwent a completely negative gastrointestinal evaluation with endoscopy, radiographic studies, and finally a surgical abdominal laparotomy. This acute abrupt onset of periumbilical pain with delayed neurological deficit was finally diagnosed as being caused by a T10 schwannoma. Abdominal symptoms can be a manifestation of a spinal lesion because of the referred pain through cross innervations between the abdominal viscera and organs with varying spinal dermatomes. Although thoracic lesions commonly present as back pain, thoracic lesions such as schwannomas must be considered in the differential diagnosis of acute abdomen without clear etiology.

## Abbreviations

GI: gastrointestinal; MRI: magnetic resonance imaging; CT: computed tomography.

## Consent

Written informed consent was obtained from the patient for publication of this case report and any accompanying images. A copy of the written consent is available for review by the Editor-in-Chief of this journal.

## Competing interests

The authors declare that they have no competing interests.

## Authors' contributions

IY analyzed and gathered the data and was a major contributor to the manuscript. EP and NH gathered data, references, analyses and contributed to the manuscript. ATP and CPA were the senior authors and provided input, reflection, and overall guidance of the manuscript. All authors read and approved the final manuscript.
